# The Human Impact of Volcanoes: a Historical Review of Events 1900-2009 and Systematic Literature Review

**DOI:** 10.1371/currents.dis.841859091a706efebf8a30f4ed7a1901

**Published:** 2013-04-16

**Authors:** Shannon Doocy, Amy Daniels, Shayna Dooling, Yuri Gorokhovich

**Affiliations:** Johns Hopkins Bloomberg School of Public Health, Baltimore, Maryland, United States; Johns Hopkins Bloomberg School of Public Health, Baltimore, Maryland, United States; Johns Hopkins Bloomberg School of Public Health, Baltimore, Maryland, United States; Lehman College, City University of New York, New York, United States

## Abstract

Introduction. More than 500 million people live within the potential exposure range of a volcano. The risk of catastrophic losses in future eruptions is significant given population growth, proximities of major cities to volcanoes, and the possibility of larger eruptions. The objectives of this review are to describe the impact of volcanoes on the human population, in terms of mortality, injury, and displacement and, to the extent possible, identify risk factors associated with these outcomes. This is one of five reviews on the human impact of natural disasters. Methods. Data on the impact of volcanoes were compiled using two methods, a historical review of volcano events from 1900 to 2009 from multiple databases and a systematic literature review of publications ending in October 2012. Analysis included descriptive statistics and bivariate tests for associations between volcano mortality and characteristics using STATA 11. Findings. There were a total of 91,789 deaths (range: 81,703-102,372), 14,068 injuries (range 11,541-17,922), and 4.72 million people affected by volcanic events between 1900 and 2008. Inconsistent reporting suggests this is an underestimate, particularly in terms of numbers injured and affected. The primary causes of mortality in recent volcanic eruptions were ash asphyxiation, thermal injuries from pyroclastic flow, and trauma. Mortality was concentrated with the ten deadliest eruptions accounting for more than 80% of deaths; 84% of fatalities occurred in four locations (the Island of Martinique (France), Colombia, Indonesia, and Guatemala). Conclusions. Changes in land use practices and population growth provide a background for increasing risk; in conjunction with increasing urbanization in at risk areas, this poses a challenge for future volcano preparedness and mitigation efforts.

## Introduction

From a global disaster perspective, volcanic eruptions result in relatively little mortality and displacement. Approximately 274,443 volcano fatalities have been documented in the historic records, with an estimated 98,386 fatalities and 5.6 million people affected in the 20^th^ century 1 ^,^ 2 . The 1902 eruption of Mount Pelee in Martinique resulted in 30,000 deaths, which is the highest number of fatalities in any 20^th^ century volcanic event. By comparison, floods were the leading cause of death in the 20^th^ century, resulting in an estimated 6.8 million deaths, and deadliest disaster of the 20^th^ century, the 1976 Tangshan earthquake, caused 242,000 deaths 3. The historical records show that the impact of volcanic eruptions on human populations is punctuated by relatively few catastrophic events with long intervals in between each event.

Approximately 9% of the global population, more than 500 million people, lives within potential exposure range of a volcano that has been active within recorded history 4 ^,^ 5 ^,^ 6. At present, there are an estimated 550 active volcanoes 7 many of which are in locations experiencing rapid population growth. Major urban centers are commonly found within close proximity to volcanoes, including Naples and the capital cities of Mexico, Japan, and the Philippines 8. Population density generally decreases as distance from the volcano increases, with the highest population densities in close proximity to volcanoes in Southeast Asia and Central America 6. The risk of catastrophic loss from future eruptions is significant given population growth, proximities of major cities to volcanoes, and the possibility of larger eruptions 9 ^,^ 10. The objectives of this review are to describe the impact of volcanoes on the human population, in terms of mortality, injury, and displacement and, to the extent possible, identify risk factors associated with these outcomes. This is one of five reviews on the human impact of natural disasters, the others being cyclones, floods, tsunamis, and earthquakes.

## Methods

Data on the impact of volcanic events were compiled using two methods, a historical review of volcanic events and a systematic literature review for publications relating to the human impacts of volcanic eruptions with a focus on mortality, injury, and displacement.

**Historical Event Review**

A historical database of significant volcanic eruptions between 1900 and 2009 was created from publicly available data. Multiple data sources were sought to ensure a complete listing of events and inclusion of both human and geophysical factors. The two primary data sources were EM-DAT: The Emergency Events Database 3 and the National Oceanic and Atmosphere Administration – National Geophysical Data Center (NOAA-NGDC) Significant Volcanic Eruption Database 11. For an event to be included in the EM-DAT database, one or more of the following criteria must be fulfilled: 10 or more people killed or injured; 100 people reported affected; declaration of a state of emergency; or a call for international assistance. In the NOAA-NGDC database, a significant eruption must meet one or more of the following criteria: caused fatalities; caused moderate damage (approximately $1 million or more); caused a tsunami; or was associated with a major earthquake.

Event lists from both databases were downloaded in July 2007 and August 2009. The reporting format for EM-DAT changed during this time period and revisions were made to some records in the NOAA-NGDC database. Event lists were reconciled to create a combined list of events for each data source; the EM-DAT list included 209 events, and the NOAA-NGDC list included 229 events. Event lists were then merged to create a complete listing of significant volcanic events between 1900 and 2009. Volcano and eruptive characteristics were abstracted from the Smithsonian Institution’s Global Volcanism Program (GVP) and added to each event 7; and data on human impacts were added from the Volcanic Disasters and Incidents Database 2. To prevent including events with no direct human impact, records where human impact (mortality, injury, or homelessness/displacement) was not quantified by any source were removed. A limitation of using the NOAA database is that events are reported if they are associated with an earthquake or tsunami, regardless of human impact. Similarly, an emergency declaration is sufficient for inclusion in the EM-DAT database, irrespective of if human populations are actually affected. The final list included 192 events reported by EM-DAT and 192 reported by NOAA; 71 events were reported by both sources yielding a total of 313 volcanic events affecting populations between 1900 and 2009. See http://www.jhsph.edu/refugee/publications_tools/index.html for the database of volcano events.

In order to examine country- and event-specific characteristics associated with low and high levels of volcano mortality, deaths were categorized as follows: low (<10 deaths), medium (11-75 deaths) and high (>75 deaths). Bivariate tests for associations between volcano characteristics and human impacts were performed using χ^2^ (categorical measures) and ANOVA (continuous measures), and a multinomial logistic regression model was used to examine the probability of evacuation in volcanic events given volcano type, time period, and certain eruptive characteristics. All analyses were performed using Stata Statistical Software, Version 11.0 12.

**Systematic Literature Review**

Key word searches in MEDLINE (Ovid Technologies, humans), EMBASE (Elsevier, B.V., humans), SCOPUS (Elsevier B.V., humans), and Web of Knowledge, Web of Science (Thomson Reuters) were performed to identify articles published in July 2007 or earlier that described natural hazards and their impact on human populations. One search was done for all the five natural hazards described in this set of papers. This paper describes the results for cyclones. The systematic review is reported according to the PRISMA guidelines. Key words used to search for natural hazards included * natural hazard(s), natural disaster(s), volcano(es), volcanic, volcanic eruption, seismic event, earthquake(s), cyclone(s), typhoon(s), hurricane(s), tropical storm(s), flood(s), flooding, mudslide(s), tsunami(s), and tidal wave(s). *Key words included for impact on human populations were**affected, damage(d), injury, injuries, injured, displaced, displacement, refugees, homeless, wounded, wound(s), death(s), mortality, casualty, casualties, killed, died, fatality, fatalities** and had to be used in either the title, abstract or as a subject heading/key word. The search resulted in 2,747 articles from MEDLINE, 3,763 articles from EMBASE, 5,219 articles from SCOPUS, and 2,285 articles from ISI Web of Knowledge. Results from the four databases were combined and duplicates were excluded to yield a total of 9,958 articles.

Title screening was performed to identify articles that were unrelated to natural disasters or human populations. Each title was screened by two independent reviewers and was retained if either or both reviewers established that inclusion criteria were met. To ensure consistent interpretation of inclusion criteria, percent agreement was assessed across reviewers for a small sample of articles, and title screening began after 80% agreement on inclusion was achieved. A total of 4,873 articles were retained for abstract review. Articles that met one or more of the following criteria were excluded in the abstract screening: language other than English; editorial or opinion letter without research-based findings; related to environmental vulnerability or hazard impact but not human populations; individual case report/study; focus on impact/perceptions of responders; and not related to human or environmental vulnerabilities or impacts of hazards. As with the title screening, overall percent agreement between reviewers was assessed and abstract screening began only after achieving 80% agreement on inclusion. Each abstract was screened by two independent reviewers and was retained if either or both reviewers established that inclusion criteria were met. During the abstract review, included abstracts were coded for event type, timeframe, region, subject of focus, and vulnerable population focus. A total of 133 articles were retained for full article review. Articles discussing the impacts of natural disasters on human populations in terms of mortality, injury, and displacement were prioritized for review. A total of 59 articles on volcanic events meeting the aforementioned subject focus criteria were retained for full review. Upon full review, 19 articles were retained including 10 that underwent dual review, standard data abstraction and 6 that were identified as review articles (Figure 1). Following the systematic review, a hand search was conducted using the databases and key words listed above to identify relevant articles published between July 2007 when the initial search was conducted and October 2012; one additional article was identified that met criteria for full review. In total, 10 articles with primary data relating to risk factors for mortality, injury or displacement were identified (Table 1) and summaries of (n=6) review articles are presented in Tables 2.

**Overview of the systematic literature review process for volcanoes:**
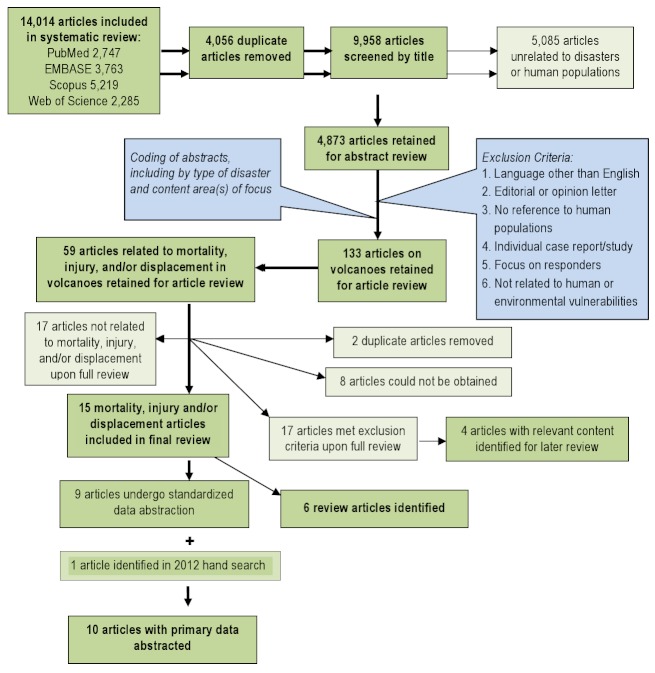

**Table 1: Articles included in the volcano systematic literature review relating to mortality, injury, and displacement (abstracted, n=10):** 

**Article**	**Event**	**Summary**	**Mortality (n=7)**	**Injury (n=4)**	**Morbidity (n=6)**	**Displacement (n=2)**
Eisele et al., 1981^16^	Mount St Helens, USA, 1980	Documents mortality attributed to the Mount St. Helens eruption	53 deaths at time of publication (35 dead, 18 presumed dead), mostly due to asphyxia; proximity was a key risk factor for mortality	Not reported	Not reported	Not reported
Merchant et al, 1982^23^	Mount St Helens, USA, 1980	Evaluates resulting health effects in surrounding areas of Washington state	32 deaths reported from asphyxia (19), burns (6), falling objects (4), fall (1), blast injury (1), and unknown (1)	Primarily respiratory problems as a result of ash exposure; also ocular irritation	Increase in emergency room respiratory admissions and respiratory conditions in high exposure groups	Not reported
Fraunfelder et al, 1983^39^	Mount St Helens, USA, 1980	Assesses ocular effects, ash exposure, and emergency room surveillance data	Not reported	Not reported	No long term ocular or respiratory effects from ash exposure; freq. of complaints increases with ash exposure	Not reported
Bernstein et al, 1986^22^	Mount St Helens, USA, 1980	Considers issues surrounding forecasting of volcanic events	48 deaths reported; among the 25 autopsied, causes of death were ash asphyxiation, (17), thermal injuries (5), trauma (3)	12 injured, from burns or ash inhalation; 72% of survivors in the damage areas injured	Respiratory effects due to ash inhalation were associated with prior lung conditions, occupation, exposure time, and distance	Not reported
Heggie et al, 2004^40^	Kilauea, USA, 1993	Describes injuries and illnesses among hikers in active lava flow areas of Volcanoes National Park	Not reported	472 injured, mostly scrapes and abrasions; novice hikers were at increased risk	Dehydration, respiratory irritation, and headaches were commonly reported	Not reported
Dent et al, 1995^41^	Rabaul, Papua New Guinea, 1994	Reports the impact of the Rabaul eruption on surrounding populations	10 deaths reported from asphyxia (3), road trauma (3), frail/ill/ stranded (2), lightning (1), flood (1)	22 injuries reported	Not reported	~ 70,000
Bourdier et al, 1997^42^	Kelut, Indonesia, 1990	Describes eruptive process; little discussion of human impact	32 deaths reported, the majority of which were due to roof collapse	Not reported	Not reported	Not reported
Horrocks, 1998^251^	Monserrat, 1995	Overview of the effects of continuing volcanic activity on Monserrat	19 deaths reported	Not reported	Not reported	~7,000 displaced; >60% resettled off the island
Grattan et al, 2003^27^	Laki Fissure, Iceland, 1783	Explores the health impact of volcanogenic gasses across Europe using historical data	Volcanogenic pollution and dry fog, and high temperatures contributed to excess mortality in England during the summer of 1783	Not reported	Difficulty breathing, eye and skin irritation, headaches, loss of appetite, and fatigue were associated with dry fog	Not reported
Carlsen et al, 2012^43^	Eyjafjallajokull 2010 eruption in Iceland	Estimates the physical and mental health effects of the Eyjafallajokull volcanic eruption on nearby residents.	Not reported	Not reported	Half of the asthmatics had more pronounced symptoms during eruption ,7% experienced post traumatic stress, Short-term ash exposure was associated with upper airway irritation.	Not reported

**Table 2: Review articles relating to volcano mortality, injury, and displacement (n=6):** 

**Article**	**Summary**	**Key Findings**
Baxter, 1986^19^	Preventative measures in volcanic eruptions	Some of the most severe eruptions have occurred without warning; explosive volcanoes are generally the most dangerous with the greatest damage occurring within a few kilometers of the volcano; fatalities will likely exceed the number of injured.
Baxter, 1998^31^	Modeling of human survival in pyroclastic flows, and factors associated with survival	Location is an important determinant of survival and injuries. Close to the eruption, conditions are unsurvivable; in distal areas, death mainly results from asphyxiation; reduced temperature and pressure are associated with increased survival.
Tanguy, 1998^14^	Historical review of mortality in volcanic eruptions between 1783-1997	An estimated 221,907 volcano-related deaths were reported in this period, including 79,286 (35.7%) from primary volcanic phenomena, most of which were associated with pyroclastic flows. Other volcano related deaths were associated with related mudflows/lahars (17.1%), tsunamis (16.9%), and famines or epidemics (30.3%).
Simpkin, 2001^1^	Review of volcanic fatalities in the historic record	More than 400 fatal volcanic eruptions have been documented in the past few decades, with an average of 2-4 fatal eruptions annually. An estimated 274,443 volcano fatalities have been documented, and there is an increasing trend in fatal eruptions which is likely associated with population growth.
Hansell, 2004^9^	Review of health hazards from volcanic gases	Studies that assess volcano related mortality suggest that volcanic gases account for less than 1-4% of mortality, though these are likely underestimates.
Witham, 2005^2^	Review of human impacts of volcanoes during the 20^th^ century	Database on deaths, injuries, evacuees and people made homeless by volcanic phenomena from 1900-1999. Includes 491 events with deaths reported in 53% of events; total death toll estimated at 91,724.

## Results

**Historical Event Review**

Overall, an average of 3 (range 1-10) volcanic eruptions affecting human populations occurred annually. When trends in reporting were assessed by source, the number of events reported annually by NOAA (range 8-27) was more consistent than EM-DAT (range 2-53), where the frequency of reported events increased over time and in particular after 1970 (Figures 2 & 3). The impact of volcanic events across regions from 1979 - 2009 is summarized in Figure 4. The World Health Organization defined regions of the Western Pacific (WPRO), Americas (AMRO) and Southeast Asia (SEARO) each accounted for more than 20% of volcanic eruptions, while both the Africa (AFRO) and the European (EURO) regions each accounted for less than 10% of eruptions; no volcanic eruptions were reported in the Eastern Mediterranean (EMRO) region. When deaths were assessed, the vast majority occurred in the AMRO region, which had 73% of reported deaths for 1900-2008; significant minorities of deaths were reported in the SEARO (16%) and WPRO regions (8%). WPRO was the region with the largest affected, which comprised approximately half of the global affected population in each time period. The AMRO region, which reported the greatest number of volcanic events and deaths for both time periods, had less than 20% of the total affected population. The overall impact of volcanic events on human populations is summarized in Table 3.

**Reporting of volcanic eruptions by source and decade:**
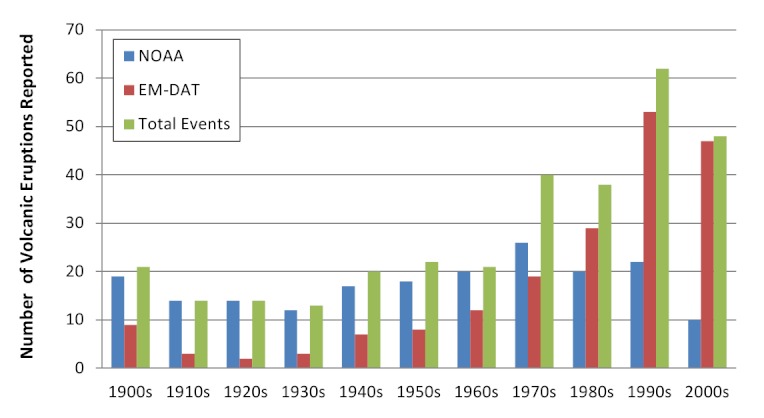

**Volcanic events affecting human populations by decade:**
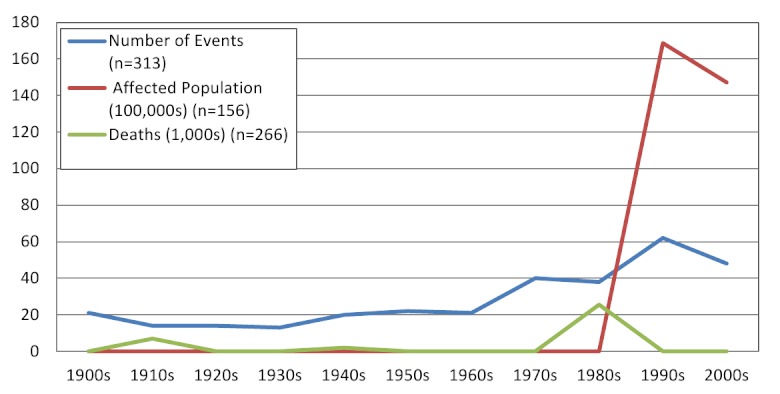

**Regional summary of volcanic eruptions and their effects on human populations,1980-2009*:**
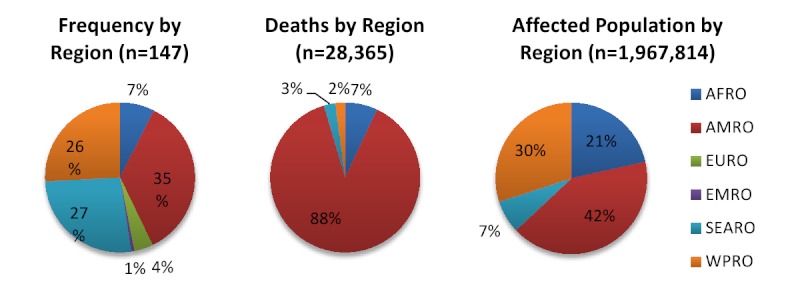
*Regions as defined by the World Health Organization

**Table 3: Summary measures for the impact of volcanoes on human populations, 1900-2009 (n=313) and 1980-2009 (n=147):** Notes: figures based on the highest reported number of deaths or injuries in an event in one country. Homeless and total affected populations are reported only by EM-DAT, thus ranges are not presented for overall impact estimates.

Cumulative Impact of Volcanoes 1900-2009 [1980-2009]								
Human Consequence	# of Events		Best Estimate		Range			
	1900-2009 (n=313)	1980-2009 (n=147)	1900-2009 (n=313)	1980-2009 (n=147)	1900-2009 (n=313)		1980-2009 (n=147)	
***Deaths***	237*	44	91,789	28,365	81,703-102,372		25,400-28,072	
***Injuries***	59	18	14,726	9,284	11,541-17,917		7,664-10,903	
***Homeless***	135	55	4,177,163	2,752,365	---		---	
***Total Affected***	156	65	4,719,414	3,128,764	---		---	
	Event Summary Statistics							
Human Consequence	# of Events		Median		Mean		Range	
	1900-2009 (n=313)	1980-2009 (n=147)	1900-2009 (n=313)	1980-2009 (n=147)	1900-2009 (n=313)	1980-2009 (n=147)	1900-2009 (n=313)	1980-2009 (n=147)
***Events with deaths***	191 [61.0%]	60 [40.8%]	6	6	536	710	1-30,000	1-23,080
EM-DAT	81 [28.0%]	35 [23.8%]	58	15	1,184	743	1-30,000	1-21,800
NOAA	163 [49.7%]	43 [29.3%]	5	4	466	681	1-28,000	23,080
***Events with Injuries***	59 [18.8%]	22 [15.0%]	11	13	304	589	1-7,500	1-10,000
EM-DAT	26 [8.3%]	18 [12.8%]	32	21	426	377	2-5,000	1-5,000
NOAA	42 [13.4%]	14 [9.5%]	6	8	325	908	1-10,000	1-10,000
***Homeless***	188 [60.1%]	106 [72.1%]	2050	5,000	20,479	32,530	0-1,035,870	450-1,035,870
***Total Affected***	156 [49.8%]	123 [83.7%]	3000	5,000	22,698	31,481	0-1,036,035	6-1,036,035

*Mortality and Injury.* When mortality data from the three sources were combined, deaths were reported in 76% (n=237) of eruptions since 1900. Overall, 91,834 deaths (range 85,169-102,372) resulting from volcanic eruptions were reported in the historical event review. For eruptions where mortality was reported, there was a median of 6 (mean = 536; 5% trimmed mean= 117; range 1-30,000) deaths per eruption when using the highest reported death toll. The ten most deadly eruptions accounted for 81.1% of reported volcano mortality (Table 4). Deaths were concentrated in Martinique (30.3% or 31,023 deaths, 3 events), Colombia (23.5% or 24,099 deaths, 7 events), Indonesia (18.4% or 18,840, 70 events) and Guatemala (12.2% or 12,522 deaths, 10 events). Injuries were reported in 59 (18.8%) events where a total of 14,726 injuries (range 11,549-17,917) were documented. In eruptions where injuries were reported, there was a median of 11 (mean = 304; 5% trimmed mean=93, range 1-10,000) injuries per eruption when the highest reported figure was used. To estimate the total number of injuries due to volcanic eruptions, it was presumed that injuries would occur in events where deaths were reported. A total of 237 eruptions with fatalities occurred were reported; when the median and 5% trimmed mean for injuries were applied to the remaining 178 events with fatalities, it was estimated that between 1,958 and 16,643 unreported volcano related injuries may have occurred between 1900 and 2008.

**Table 4: The ten deadliest volcanic eruptions between 1900 and 2009:** Notes: table includes the maximum number of deaths reported for the event. Percent calculated based on maximum reported deaths in all volcanic events between 1900 and 2009.

	**Deaths Reported**	**Percent**	**Injured**	**Total Affected**
1. Mount Pelee (Martinique, 1902)	30,000	29.3%	Not reported	Not reported
2. Nevado del Ruiz (Colombia, 1985)	23,080	22.5%	10,000	12,700
3. Santa Maria (Guatemala, 1902)	6,000	5.9%	Not reported	Not reported
4. Semeru (Indonesia, 1909)	5,500	5.4%	Not reported	Not reported
5. Kelut (Indonesia, 1919)	5,110	5.0%	Not reported	Not reported
6. Santa Maria (Guatemala, 1929)	5,000	4.9%	Not reported	Not reported
7. Lamington (Papua New Guinea, 1951)	3,000	2.9%	Not reported	Not reported
8. El Chichon (Mexico, 1982)	1,879	1.8%	500	40,500
9. Oku Volcanic Field (Cameroon, 1986)	1,746	1.7%	437	10,437
10. Soufriere Hills (St Vincent, 1902)	1,680	1.6%	Not reported	Not reported
**Total**	**82,995**	**81.1%**		

Data to compute ratios of deaths to injured and affected populations was available for 58 and 130 historical events, respectively. Wide variation in ratios was observed, presumably because of variation in reporting quality. For 1900-2008, the median ratio of dead: injured was 0.63 (mean 2.53, range 0-36). When compared to the affected population, there was a median of 0 deaths per 1000 affected (mean = 96, range 0-6709). Point estimates for both ratios decreased by approximately half in the period of 1980 to 2008, which may suggest improvements in disaster response and preparedness; however, these changes were not statistically significant. Multinomial logistic regression for predictors of evacuation indicates that time period of the eruption (among other factors) is significantly associated with evacuation, where eruptions occurring in 1980 and later were 20.25 (CI: 8.01-51.21) more likely to have an evacuation reported as compared to those between 1900-1939, which also suggests improvements in preparedness and response (Table 5).

**Table 5: Logistic regression model for evacuation in volcanic events (n=293):** Note: based on a forward multinomial logistic model including all variables in Table 5.

	Coefficient (95 CI)		p-value
VEI	1.71 (1.22-2.39)		.002
Volcano type			
*Complex volcano*	Reference		
*Shield volcano*	3.78 (0.96-16.3)		.056
*Stratovolcano*	4.52 (1.76-11.62)		.002
*Other*	5.60 (1.37-22.94)		.017
Time Period			
*1900-1939*	Reference		
*1940-1979*	3.78 (1.59-8.93)		.002
*1980-2008*	20.25 (8.01-51.21)		<.001
Pyroclastic Flow	2.61 (1.28-5.34)		.008
Flank Vent	3.95 (1.82-8.62)		<.001
Mudflows	2.54 (1.25-5.17)		.010
Lava Dome	1.96 (0.93-4.13)		.075
**Model Statistics**	80.5% Predicted Correct		
HL Goodness of Fit	X^2^ = 123, df=9	<.001	

*Affected Population and Other Impacts.* An estimated 4.7 million people were reported to be affected by volcanoes between 1900 and 2009, including 4.2 million that were rendered homeless. However, these figures are likely to substantially underestimate the true impact of volcanoes on human populations because estimates of the total affected population and the homeless population were reported in 49.8% (n=156) and 43.3% (n=135) of events, respectively. The distribution of the affected population was highly skewed: on average 22,698 people were affected in an eruption, though the median affected population was significantly lower at 3,000. Other measures of effect on human populations reported by GVP included evacuation and damages in excess of one million dollars; these data were reported for 296 (94.6%) of events. Damage in excess of one million dollars were reported in 75.0% of events (n=222/296) and evacuation was reported in 56.5% (n=177/296) of events.

*Eruptive Characteristics.* Eruptive characteristics were reported by GVP for 296 (94.6%) eruptions in the event file. When assessed by volcano type, proportions were as follows: stratovolcano 73% (n=228), complex volcano 13% (n=39); shield volcano 7% (n=22); caldera 4% (n=11); cinder cone 2% (n=4); and others 2% (n=4). VEI is a logarithmic scale (0-8) indicating the amount of mass ejected during an eruption and the size of the eruption column. 13 In general, VEI 1-3 events generate localized hazardous phenomena and VEI 4-5 events have the potential for disruption on a regional scale; VEI 6+ events can affect the entire planet via their impact on global climate 10. The average VEI of eruptions was 2.60 (Table 6). In a multinomial regression model, an average of 362 additional deaths (CI: 57-667, p=.020) were associated with a one unit increase in VEI after controlling for evacuation.

**Table 6: Human impacts by volcano and eruptive characteristics, 1900-2009*:** *mean affected population reported by EM-DAT; damage and evacuation reported by GVP

	Frequency of Events	Events with Deaths	Mean Deaths (median)	Mean Affected, Thousands (median)	Damage> 1M USD	Evacuation Reported
***Volcano Type(n=312)***						
Complex volcano	39 (12.5%)	87.20%	19 (10)	7.9 (3.0)	71.40%	31.40%
Shield volcano	22 (7.1%)	77.30%	121 (2.5)	27.9 (2.5)	55.60%	38.90%
Stratovolcano	228 (73.1%)	72.10%	482 (10)	37.0 (10.1)	78.50%	65.10%
Others	23 (7.4%)	66.70%	137 (419)	6.41 (4.6)	84.20%	57.90%
***VEI (n=273)***						
1 or less	29 (10.6%)	72.40%	37 (95)	21.4 (5.3)	44.80%	44.80%
2	104 (38.1%)	69.20%	83 (30)	24.8 (4.9)	69.20%	42.30%
3	100 (36.6%)	81.00%	403 (32)	18.8 (5.5)	84.00%	71.00%
4 or more	40 (14.7%)	87.50%	1523(500)	57.1 (40.5)	97.50%	77.50%
***Eruptive Characteristics***						
Central Vent	226 (80.4%)	77.00%	416 (42)	40.2 (3)	78.20%	60.60%
No Central Vent	55 (17.4%)	78.20%	305 (158)	9.9 (6)	76.10%	50.90%
Pyroclastic Flow	152 (54.3%)	82.90%	103 (37)	20.5 (47.5)	88.20%	73.70%
No pyroclastic Flow	128 (45.7%)	70.30%	645 (58)	44.9 (7.7)	62.50%	40.60%
Lava Flow	137 (48.8%)	75.90%	101 (38.5)	34.6 (7.7)	78.80%	65.00%
No Lava Flow	144 (51.2%)	78.50%	679 (89)	36.4 (5.1)	74.30%	52.80%
Lava Dome	80 (28.5%)	82.50%	613 (37)	49.6 (5.0)	82.50%	78.80%
No Lava Dome	201 (71.5%)	75.10%	308 (100)	29.6 (7.1)	74.10%	50.70%
Flank Vent	61 (21.7%)	83.60%	807 (175)	70.6 (6.9)	85.20%	75.40%
No Flank Vent	220 (78.3%)	75.50%	273 (32)	25.3 (6)	74.10%	54.10%
Radial Fissure	63 (22.4%)	73.00%	81 (56.5)	22.9 (5.9)	84.10%	61.90%
No Radial Fissure	218 (77.6%)	78.40%	483 (68.5)	38.9 (6.0)	74.30%	57.80%
Phreatic Explosion	105 (37.4%)	80.00%	766 (90)	43.0 (6.3)	79.00%	70.50%
No Explosion	176 (62.6%)	75.60%	149 (58)	30.1 (6.0)	75.00%	51.70%
Crater Lake Eruption	22 (7.8%)	72.70%	2073(677.5)	19.9 (10.2)	77.30%	50.00%
No Lake Eruption	259 (92.2%)	77.60%	252 (41.5)	36.6 (6.0)	76.40%	59.50%
Debris Avalanche	27 (9.6%)	81.50%	440 (58)	3.7 (4.0)	88.90%	66.70%
No Avalanche	254 (90.4%)	76.80%	390 (79)	38.4 (7.8)	75.20%	57.90%
Mudflow	126 (44.8%)	87.30%	758 (84)	55.3 (9.0)	89.70%	74.60%
No mudflow	155 (55.2%)	69.00%	94 (30)	17.6 (5.6)	65.80%	45.80%
Tsunami	27 (9.6%)	81.50%	2015(431)	26.4 (5)	77.80%	48.10%
No tsunami	254 (90.4%)	76.80%	271 (48)	36.3 (6.7)	76.40%	59.80%

***Systematic Literature Review***

*Mortality.* A review by Tanguy (1998) estimates 221,907 deaths from major volcanic eruptions over the past two centuries (1783-1997), which includes 90% of recorded deaths throughout history. Volcano related mortality was attributed to primary volcanic phenomena (see table 7 for definitions) including ash and pyrolcastic flows (36%), mudflows or lahars (17.1%), volcanogenic tsunamis (16.9%), and post-eruption epidemics or famines (30.3%). Of the 79,286 deaths due to primary volcanic phenomena, 75.0% were attributed to pyroclastic flows or surges and magma-generated sector collapse, followed by debris avalanches (12.6%), ash fall including ballistic projectiles (11.6%), and lava flows (0.8%). Four volcanic eruptions (Tambora, 1815; Krakatau, 1883; Mt. Pelee, 1902; and Ruiz, 1985) accounted for more than 66% of deaths, and the principal causes of death, including famine, tsunamis, pyroclastic flows, and lahars varied in each event. While famine and epidemics were the principal causes of mortality in earlier volcanic events, they currently present much less of a risk due in large part to international relief and humanitarian assistance efforts 14.

**Table 7: Volcanic Hazards and Mitigation Strategies:** Source: adapted from McGuire, 1998 unless otherwise cited; excludes the related hazards of volcano-related earthquakes and tsunamis.

**Pyroclastic flows** and surges are among the most life-threatening of volcanogenic hazards; high velocities combined with extreme temperatures can lead to severe destruction.	Prior evacuation of threatened areas (identified by hazard mapping) can reduce loss of life. More broadly, effects of pyroclastic flows could be mitigated via long-term urban planning (subways, strong buildings) in lieu of evacuations plans (which are often difficult to implement).^14^
**Lava flows** are driven by gravity and their paths are constrained by topography and can thus be predicted.	Hazard maps can be developed for areas of at risk of flow-related damage; more advanced models incorporate digital terrain mapping and solidification during advance which allows for rapid prediction of flow-field volume.^44^
**Debris flows (lahars)** and floods are responsible for nearly all of the volcano-related deaths in the last two decades of the 20^th^ century as well as major destructive impacts on surrounding communities.	Lahars are highly topographically constrained, and commonly are confined to river courses, allowing for the prediction of likely debris flow paths, hazard mapping, and identification of high risk areas. Warning systems that permit evacuation could reduce the lethal effect of lahars. Engineering measures, such construction of baffles and sediment dams, can reduce flow mass and resulting destruction. Long-term urban planning can also reduce risk by avoidance of dense population concentrations in valleys and deltas.^14^
**Tephra** can be one of the most disruptive volcanic hazards, with ash fall typically representing the most extensive and disruptive form.	In the developing world, tephra accumulation on roofs of poorly constructed buildings often results in collapse; campaigns to promote ash removal can reduce fatalities and damage from tephra-induced roof collapse. Humanitarian assistance can reduce potential long-term impacts of resulting crop loss, livestock deaths, and economic disruption.
**Debris avalanches (volcanic landslides)** may be associated with an eruption but also occur during periods of inactivity.^45^	High velocities make prior evacuation the only measure for preventing loss of life. However, evacuation can present a challenge because it requires recognition that a landslide is likely in the short term and identification of the threatened areas.
**Gas** emissions associated with eruptions or degassing activity include sulphur-dioxide, sulphuric acid, aerosol, hydrochloric acid, carbon dioxide, hydrogen sulfide, mercury, radon and other gases.^8^	Reductions of future morbidity associated with volcanic gas include identification and ongoing monitoring of hazardous lakes, resettlement, pre-evacuation, and establishing guidelines relative to areas of refuge.^46^

Another database developed by Witham (2005) considers all volcano-related incidents (n=491) with human impacts between 1900 and 1999, including 296 (60%), which were classified as disasters. Witham found the number of people impacted per event was substantially greater in middle income than in high income countries, with middle income countries reporting the highest numbers of volcano related deaths, injuries, and affected populations 2. While fatalities were the most frequent outcome, observed in 53% of events, the largest consequence was displacement, which accounted for 94% of the affected population. Similar to historic findings, the study found fatalities to be concentrated in relatively few large events. The eruptions of Mt. Pelee (1902) and Nevado del Ruiz (1985), which together accounted for more than 50% of deaths, and the top ten events combine to account for more than 90% of deaths. During the 20^th^ century, pyroclastic flows were the primary cause of death followed by lahars (which were also the principal cause of injury), and tephra was a primary cause for evacuation and displacement 2. Tephra, which includes fragmental material from volcanoes and volcanic ash, is also a common cause of trauma-related deaths either by projectile impact or collapse of ash-covered roofs 1. The three main causes of direct mortality in recent volcanic eruptions include asphyxiation from ash, thermal injuries from pyroclastic flow, and trauma (Table 2) 15 ^,^ 16. A review of health hazards from volcanic gases indicates that volcanic gases account for less than 1% to 4% of mortality, though this is a likely underestimate because studies neglect volcanic degassing when unassociated with eruptive activity, and because the extent of dispersion of volcanic gases is not always appreciated 17. Historical estimates in the 20^th^ Century suggest that 2,000 deaths have resulted from volcanic gases, with the most hazardous volcanogenic gases being carbon dioxide, sulfur dioxide, hydrogen sulfide, mercury, and radon 18.

*Morbidity.* Transient increases in trauma-related injuries resulting from traffic accidents and falls, and morbidity, primarily ocular irritations and respiratory symptoms, are observed following volcanic eruptions. Increases in communicable diseases and long-term health effects are not attributed to volcanic eruptions, however, volcanism associated morbidity is likely underestimated 9. Ash fall can have health implications for populations as far as hundreds of kilometers away 19. A recent review of the respiratory effects of volcanic ash suggests that they are short-lived and dependent on the mode of ash generation and particle size, among other factors 20. De novo appearance of ash-related asthma has not been documented, though increases in respiratory symptoms are frequently reported. Higher levels of ash exposure have been associated with increased reports of respiratory symptoms among children 21 and in high-exposure occupation groups 22 ^,^ 23. Increases in emergency room visits for respiratory problems have been observed following recent volcanic eruptions in the United States and Ecuador, where children under five 24 and individuals with pre-existing lung conditions such as asthma or chronic bronchitis were at increased risk for the development of respiratory symptoms 20 ^,^ 22 ^,^ 23. Recently, adverse cardiorespiratory health effects have been associated with chronic exposure to volcanogenic gases in both Hawaii and Montserrat 25 ^,^ 26 and historically, where one study suggested that adverse health symptoms in Europe during the summer of 1783 was associated with volcanic gases and fogs 27. Review findings indicate that volcanic eruptions are associated with short-term increases in morbidity, primarily ocular irritations and respiratory symptoms.

## Discussion

***Main Findings***

An increasing trend in the total population affected by volcanoes each decade is observed after 1950; however, this is not unexpected considering this information is reported by EM-DAT where the number of events reported increased over time. Other factors likely contributing to this observation are improved data reporting quality and population growth, which has resulted in a larger population and greater levels of development in at risk areas. This is poses concerns, where lower magnitude eruptions in areas that have experienced significant land use change and high population growth may have greater impacts than anticipated when projections are based on the historical record alone. The 1985 eruption of the Colombian volcano Nevado Del Ruiz is an example of increasing human vulnerability to volcanoes due of population growth in high-risk areas. The town of Armero, which was completely buried by lahars in 1985, experienced similar lahars in 1595 and 1845 and in both instances the community was rebuilt and population expanded 28. In addition to population growth and land use change, globalization is an emerging factor, which may contribute also to increased vulnerability to natural disasters and result in consequences that span larger geographic regions 29.

The increasing frequency of volcanic incidents and impacts on human populations has been documented elsewhere and may reflect numerous factors, including increased reporting, increased use of evacuation in risk mitigation, growth in the population at risk, and actual changes in global volcanic activity 1 ^,^ 2. A total of 102,373 volcano related deaths and an average of 430 deaths per eruption were reported between 1900 and 2008 when the highest mortality estimate from any source in the historical event review was used (102,140 deaths were reported during the 20^th^ century). Other estimates of 20^th^ century volcano mortality range from 91,724 to 98,376, with an average of 845 to 917 deaths per eruption 2 ^,^ 14. Mortality was concentrated in several significant eruptions, most notably the 1902 eruption of Mt. Pelee on the Island of Martinique which resulted in 28,000 to 30,000 deaths and the 1985 eruption of Colombia’s Nevado Del Ruiz where a lahar caused an estimated 21,800 to 23,080 fatalities. Overall, 45% of volcanic deaths between 1900 and 2008 were reported in the 1900s, 29% in the 1980s, and the remaining 26% in other decades.

Historically, pyroclastic flows have accounted for the majority of mortality in volcanic events, which suggests they will continue to be the most lethal volcanic agent in the near future. It has been widely assumed that pyroclastic flows are unsurvivable, but evidence from eruptions in the 20^th^ century challenges this assumption, especially at the periphery of flows where impacts are attenuated and protection can be provided by resistant buildings 30. Modeling and reviews of volcanic deaths and injuries in pyroclastic flows from recent decades suggests that survival is possible under certain conditions. Survival limits on heat exposure, inhalation of hot air, and air containing hot particles have been established, and simulations show that in distal areas survival is possible. Close to the volcanic crater, conditions are unsurvivable due to heat, high particle concentrations, and elevated dynamic pressures. In distal areas of pyroclastic flows, death and injury are mainly related to asphyxiating levels of particles; reduced temperature and lower dynamic pressures increase the probability of survival, especially in masonry buildings which should be the most resistant to the impact of pyroclastic flows 31.

Since 1980, volcanic disasters have resulted in nearly 30,000 deaths, two-thirds of which were preventable and associated with a single event (Nevado del Ruiz, 1985) which suggests that improved mitigation measures have the potential to reduce loss of life in future eruptions. Effective communication with civil authorities and vulnerable populations and education about the threats posed by volcanoes is equally important as volcano monitoring and forecasting. Initiation of volcano awareness programs in volcanic hazards prone areas and contingency planning are also of central importance, particularly as the size of the population living close to active volcanoes increases 10. Accurate forecasting of the eruption force and prediction of its occurrence by volcanologists and timely evacuation of populations at risk are vital to effective emergency management, and risk assessment is an integral part of disaster preparedness 31. Monitoring has a critical role to play in reducing the impact of volcanic hazards by providing early warning and possibly identifying timeframes of forthcoming eruptions 10. In 1994, just more than 20% of the approximately 550 active volcanoes were monitored, and the extent to which monitoring has increased remains unclear 7. The crucial role of monitoring volcanic activity was exemplified during eruptive activities of Mt. St. Helens and Pinatubo when early warning signs were evident enough to start evacuation of population, involve emergency services, which ultimately minimized the number of victims.

While a wide range of geophysical, geochemical and geodetic techniques are available 32 ^,^ 33 the ‘core methods’ of seismic and ground deformation monitoring are the best means of tracking magma movement and accumulation. Increased monitoring, either via satellite platforms capable of detecting pre-eruptive ground deformation 34 and thermal anomalies 35 or ground monitoring is of vital importance. Geodetic and oceanographic surveys of active submerged volcanic cones have recently been employed to assess the state of their hydrothermal activity, sample gases and volcanic rocks 36 ^,^ 37. Periodic assessment of changes in topography of submerged volcanic cones and their geochemical activity will provide early warning signs of possible phreatic explosions. Comprehensive monitoring of volcanoes in conjunction with hazard zonation maps offer the best means of reducing casualties, primarily through ensuring evacuation of people from the threatened areas 10. Hazard zonation maps show areas of potential volcanic impacts, such as possible propagation of pyroclastic or lava flows, locations of eruptive centers, areas of probable landslides, tsunami effects and ash fallout 38. These maps are invaluable tools in planning of mitigation measures (Table 7). Evidence from recent eruptions suggests that emergency planning for explosive eruptions in urban areas should concentrate on distal areas of predicted pyroclastic flows and areas where the primary risk is death due to asphyxiation from ash inhalation rather than death due to injury. This includes the potential need to rescue survivors suffering from ash inhalation as well as trauma; a smaller number of victims may require treatment for dermal and airway burns, though their numbers may easily exceed regional capacity for treatment 31.

***Limitations***

Systematic reviews face numerous limitations. The effects of volcanic eruptions are the subject of gross approximations and aggregations that have a great deal of imprecision. The availability and quality of data has likely increased and improved over time, however, in many events deaths are unknown or unrecorded. For a significant number of events no data are reported for injured, displaced, and affected populations; this likely contributes to a substantial underestimation of the impacts of volcanoes on human populations. Inconsistencies and errors were common in data files from different sources, and in some cases inclusion criteria were not ideal for the purposes of this review, which created a challenge in reconciling event lists. When combined with the relatively small number of recent volcanic events, uncertainty in the historical record and the relative paucity of primary research focusing on the health-related topics significantly limits the conclusions that can be drawn about volcanic impacts on human populations. A principal limitation of the literature review is the fact that only English language publications were included; this likely contributed to incomplete coverage of studies published in other languages originating from low and middle income countries

## Conclusions

The impact of volcanoes on humans in terms of mortality, injury, and affected populations, presented here is a minimum estimate because information for many volcanic events is either unknown or unreported. Data from 1900 to the present suggest that volcanoes have exacted a relatively small toll on the human population when compared to other natural disasters. However, human vulnerability to volcanic hazards is increasing, in large part due to land use change and particularly to the development of densely populated urban areas in close proximity to volcanoes.

Medical treatment clearly has a limited role in volcanic eruptions because severe injuries occur only in a relatively concentrated area, and morbidity experienced in more outlying areas is limited in both severity and duration. A strong emphasis on preparedness strategies is required, and because many major volcanic eruptions are preceded by warning signs, it is possible to plan for these events, which is evident given the increasing trend in evacuations. Because volcano fatalities are concentrated geographically in relatively few eruptions, targeted preparedness efforts in areas that are historically at risk as well as those newly identified via monitoring could be successful. Hazard evaluations for all volcanoes in populated areas, regardless of their active or dormant state, and expanded monitoring could improve preparedness levels. Hazard-specific mitigation strategies such as engineering projects or urban planning could be implemented to reduce potential impacts; however, their costs may be prohibitive when compared to the likelihood of an eruption in the near future. Broader-based awareness and education strategies targeted at the population at risk would likely result in more successful evacuations and may also increase willingness of authorities to implement more costly preparedness measures.

## Competing Interests

The authors have declared that no competing interests exist.

## Correspondence

Shannon Doocy, Johns Hopkins Bloomberg School of Public Health, 615 N. Wolfe St, Suite E8132, Baltimore, MD 21230. Tel. 410-502-2628. Fax: 410-614-1419. Email: sdoocy@jhsph.edu.

## Appendix 1

### PRISMA Checklist

Download PDF
